# Lemon Flavonoid Extract Eriomin Improves Pro/Antioxidant Status and Interferes with Cholesterol Metabolism without Affecting Serum Cholesterol Levels in Aged Rats

**DOI:** 10.3390/ijms25105221

**Published:** 2024-05-10

**Authors:** Branka Šošić-Jurjević, Slavica Borković-Mitić, Slađan Pavlović, Dragana Vlahović, Marko Miler, Thais Cesar, Vladimir Ajdžanović, Dragan Milenkovic, Frans Stellaard, Svetlana Trifunović, Branko Filipović, Dieter Lütjohann

**Affiliations:** 1 Institute for Biological Research “Siniša Stanković”—National Institute of Republic of Serbia, University of Belgrade, Bulevar despota Stefana 142, 11108 Belgrade, Serbia; borkos@ibiss.bg.ac.rs (S.B.-M.); sladjan@ibiss.bg.ac.rs (S.P.); dragana.vlahovic@ibiss.bg.ac.rs (D.V.); marko.miler@ibiss.bg.ac.rs (M.M.); avlada@ibiss.bg.ac.rs (V.A.); lanat@ibiss.bg.ac.rs (S.T.); brankof@ibiss.bg.ac.rs (B.F.); 2 Graduate Program in Food, Nutrition and Food Engineering, Sao Paulo State University (UNESP), Araraquara 14800-060, Brazil; thais.cesar@unesp.br; 3 Department of Nutrition, University of California Davis, Davis, CA 95616, USA; dmilenkovic@ucdavis.edu; 4 Institute of Clinical Chemistry and Clinical Pharmacology, University Hospital Bonn, Venusberg-Campus 1, 53127 Bonn, Germany; fstellaard@hotmail.com (F.S.); dieter.luetjohann@ukbonn.de (D.L.)

**Keywords:** lemon flavonoids, Eriomin, aging, rat, liver, oxidative stress, cholesterol, desmosterol, 7α-hydroxycholesterol, CYP7A1

## Abstract

This study aimed to assess the antioxidant capacity of lemon flavonoid extract Eriomin^®^ (LE) and its impact on cholesterol metabolism in the context of healthy aging. We orally treated 24-month-old male Wistar rats with an LE (40 mg/kg) suspended in 0.3 mL of sunflower oil. At the same time, control groups received an equal volume of sunflower oil (CON) or remained untreated (ICON) daily for 4 weeks. We examined LE’s effects on superoxide dismutase and catalase- and glutathione-related enzyme activities, the concentration of lipid peroxides and protein carbonyls, total oxidant status (TOS) and antioxidant status (TAS), and oxidative stress index (OSI) in the liver, jejunum, and ileum. We also measured total cholesterol, its biosynthetic precursors (lanosterol, lathosterol, desmosterol), its degradation products (bile acid precursors) in the serum, liver, jejunum, and ileum, and serum phytosterols (intestinal absorption markers). LE reduced TOS, TAS, and OSI (*p* < 0.05) compared with control values, indicating its consistent antioxidant action in all examined organs. LE lowered hepatic desmosterol (*p* < 0.05) while also reducing 7α- and 24-hydroxycholesterol levels in the liver and ileum (*p* < 0.01). Serum cholesterol, hepatic gene expression, and the immunostaining intensity of CYP7A1 were unchanged. In conclusion, LE exerted non-enzymatic antioxidant effects and reduced cholesterol degradation, reducing its biosynthesis products, thereby maintaining serum cholesterol levels.

## 1. Introduction

Cholesterol homeostasis, which is crucial for the maintenance of health, relies on the delicate balance between dietary cholesterol absorption, de novo synthesis, and biliary secretion and elimination [1]. Disruption of this balance can result in elevated cholesterol levels and hypercholesterolemia, which is common in the elderly and is influenced by lifestyle and genetic factors [2]. This population is particularly susceptible to a significant burden of cardiovascular disease and mortality, with a known association between elevated plasma cholesterol and coronary heart disease and non-alcoholic fatty liver disease in older individuals [2,3]. On the other hand, citrus flavonoids have been reported to have cardioprotective, hepatoprotective, hypolipidemic, and antidiabetic effects related to their antioxidant activity [4,5]. Their positive effect on reducing the risk of cardiovascular disease was confirmed in a randomized, double-blind, placebo-controlled study in healthy volunteers [6]. Among the diverse group of citrus flavonoids, eriocitrin (eriodictyol 7-O-β-rutinoside), the primary bioactive compound in lemon extract, is the least studied in current research [7]. During the aging process, there is an elevation in reactive oxidative species (ROS) production, together with a decline in the antioxidant defense in the liver and small intestine [8,9]. Elevated ROS levels can cause oxidative stress and severe damage to the cell, organelle membranes, DNA, lipids, and proteins and interferes with the body’s metabolic activity [8,10]. In the early stages, age-related changes may be prevented by exposure to chemopreventive compounds, such as nutritional factors, particularly in the liver, which has a high regenerative capacity [8,11]. Using eriocitrin to attenuate oxidative stress, which has been demonstrated in crossover-randomized clinical trials for metabolic diseases such as diabetes mellitus, and has the potential to reduce systemic inflammation in obesity models and other chronic diseases [12,13]. In addition, eriocitrin has been reported to reduce diet-induced hepatic steatosis [14]. Therefore, there is a need for further studies on its metabolic effects and potential therapeutic applications.

The liver and small intestine, mainly the jejunum and ileum, are the main organs that control cholesterol homeostasis. The small intestine is second only to the liver in its contribution to endogenous cholesterol biosynthesis. Additionally, the proximal small intestine is primarily responsible for cholesterol absorption, while the distal ileum actively reabsorbs bile acids, which then return to the liver through the enterohepatic circulation [15]. This process inhibits cholesterol biosynthesis and further bile acid biosynthesis. The underlying mechanism(s) of the age-related increase in serum cholesterol in rodent models [16,17] seems to be sex-specific: in males, it has been related to reduced hepatic degradation, decreased hepatic cholesterol 7-hydroxylase A1 (CYP7A1) activity, and elimination of cholesterol [17,18], while in females, it seems to be mainly due to increased intestinal cholesterol absorption [9,19].

Recent studies from our group on the effects of the lemon flavonoid extract Eriomin^®^ (containing 70% eriocitrin in combination with other citrus flavonoids; LE) on the livers of aged rats have shown an increase in gene and protein expression, as well as nuclear localization of the nuclear factor erythroid 2-related factor 2 (Nrf2) along with an increase in the protein expression of thioredoxin 1 (Trx1) [7]. These molecular changes may contribute to the strong antioxidant potential of citrus flavonoids. In addition, the Nrf2 signaling pathway has been reported to be involved in the regulation of CYP450 enzymes involved in cholesterol and bile acid metabolism: Nrf2 activation has been associated with a decrease in hepatic CYP7A1, the rate-limiting enzyme in the main (neutral) pathway of cholesterol degradation to bile acids, which may affect the elimination of cholesterol in vitro and in vivo [20,21]. However, polyphenols such as resveratrol, puerarin, and quercetin have been associated with the induction of CYP7A1 [14].

The concept of healthy aging is strongly supported by the World Health Organization due to the demographic situation and the increasing older population [22]. People in this age group typically experience a functional decline in the gastrointestinal system and metabolic regulation. These problems may affect their quality of life and increase their susceptibility to the adverse effects of cholesterol-regulating medications, making them potentially the most important beneficiaries of supplementation. Data on the effects of citrus flavonoids on older adults are limited. 

This study aims to improve our understanding of the antioxidant capacity of lemon extract and its cholesterol-reducing potential in the context of apparent healthy aging. To address this goal, we performed a comprehensive analysis of oxidative stress parameters, including: (i) the antioxidative defense system; and (ii) oxidative stress-induced damage, as well as pro/antioxidant status in the liver and small intestine, namely jejunum and ileum. Furthermore, changes in cholesterol metabolism are assessed by measuring: (i) total cholesterol, (ii) surrogate markers of cholesterol biosynthesis, and (iii) key cholesterol degradation products to bile acids in the serum, liver, and small intestine (jejunum and ileum), as well as (iv) surrogate cholesterol absorption markers (phytosterols: campesterol, sitosterol, and stigmasterol) in serum. Particular emphasis is placed on the analysis of the expression of CYP7A1 as a possible target of the Nrf-2-mediated effect of the lemon extract. To the best of our knowledge, such a comprehensive study has not yet been performed.

## 2. Results

The daily food intake, body mass, and absolute and relative liver weight of all experimental groups are summarized in Table 1. The body weight values obtained for the ICON group are within the standard values for the corresponding age of Wistar rats. Wistar rats are an outbred strain characterized by a spontaneous increase in body weight throughout life when fed ad libitum with standard laboratory chow. This characteristic could make them a model for spontaneous mild obesity. The comparison between the different treatment groups ICON, CON, and LE revealed no significant differences in food intake, body mass, or absolute and relative liver mass (Table 1). 

### 2.1. Effects of Lemon Extract on Oxidative Stress Parameters 

Table 2 lists all parameters of oxidative stress measured in the liver, jejunum, and ileum. 

No significant differences were found in the activities of antioxidant defense enzymes in all examined organs among experimental groups. 

Comparisons between ICON and CON revealed that sunflower oil exerted tissue-specific effects on the examined oxidative stress parameters. The smallest overall impact was observed in the liver: sunflower oil increased GSH and sulfhydryl group (SH) concentrations (*p* < 0.05). The greatest impact was observed in the jejunum: it reduced GSH, TOS, and OSI (*p* < 0.05) while increasing lipid peroxidation products (LPO; *p* < 0.01) and TAS (*p* < 0.05). In the ileum, it increased GSH (*p* < 0.05) and OSI (*p* < 0.01) while reducing TAS (*p* < 0.001).

In the jejunum, LPO was decreased (*p* < 0.01) in the LE group compared with the values obtained for CON group, indicating lower oxidative degradation of cellular lipids. The levels of TOS, TAS, and OSI were consistently reduced in all examined organs (*p* < 0.05 or lower; Table 2) in the LE group compared with the CON group. 

Data on multivariate tests of significance, including *p*-values, effect sizes, and powers for all oxidative stress parameters are presented in the Supplementary Materials (Tables S1 and S2).

### 2.2. Effects of Lemon Extract on the Parameters of Cholesterol Metabolism 

**Figure 1:**
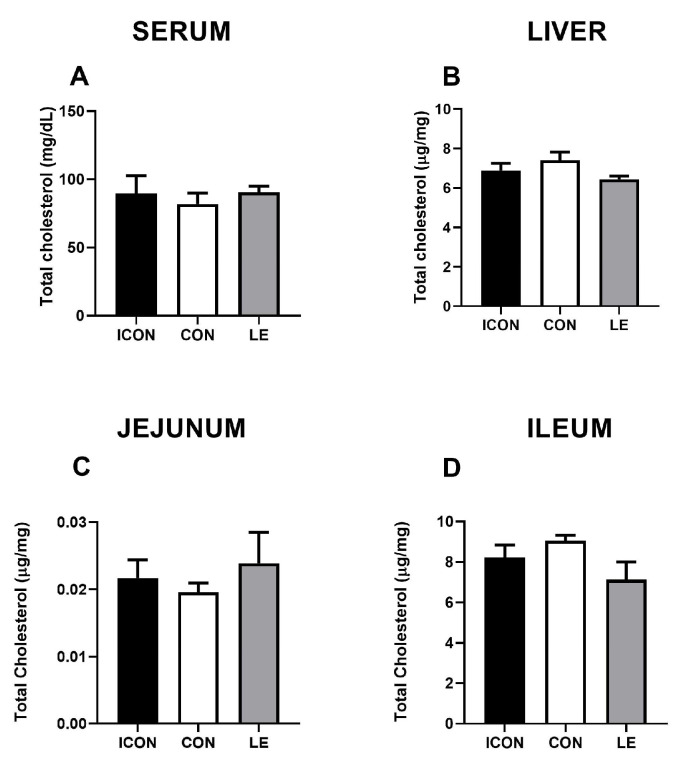
Effects of lemon flavonoid extract Eriomin^®^ (LE) on the concentration of total cholesterol in the serum (**A**), liver (**B**), jejunum (**C**), and ileum (**D**) in old-aged male rats. The groups are abbreviated as intact control (ICON), sunflower oil-treated control (CON), and lemon extract-treated (LE). Data are presented as the mean ± SEM (n = 7/group); comparisons between the CON and the study groups were not statistically significant (one-way ANOVA test followed by Dunnet’s post hoc test).

Statistical analysis revealed no significant differences in total cholesterol concentrations in the serum, liver, and small intestine amongst the experimental groups (Figure 1A–D). Data on multivariate tests of significance, including *p*-values, effect sizes, and power, for all measured parameters of cholesterol metabolism are presented in the Supplementary Materials (Tables S1 and S2). 

**Figure 2:**
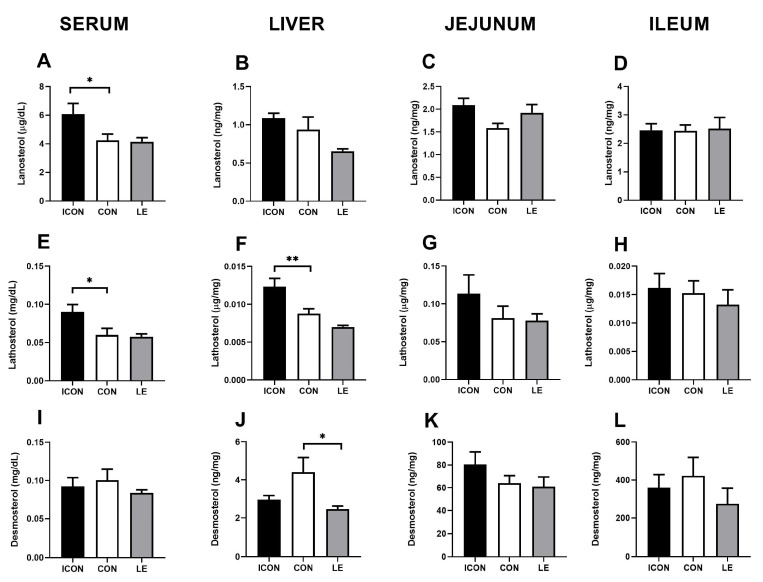
Effects of lemon flavonoid extract Eriomin^®^ (LE) on the concentration of lanosterol (**A**–**D**), lathosterol (**E**–**H**), and desmosterol (**I**–**L**) in the serum, liver, jejunum, and ileum in old-aged male rats. The groups are abbreviated as intact control (ICON), sunflower oil-treated control (CON), and lemon extract-treated (LE). Data are presented as the mean ± SEM (n = 7/group); one-way ANOVA test followed by Dunnet’s post hoc test (CON served as the control group); * *p* < 0.05, ** *p* < 0.01; the remaining comparisons between CON and the study groups were not statistically significant.

To further investigate the putative effects of LE on cholesterol biosynthesis, we measured the cholesterol precursors (surrogate synthesis markers) lanosterol, lathosterol, and desmosterol in the serum, liver, jejunum, and ileum (Figure 2A–L). Sunflower oil significantly reduced (*p* < 0.05) concentrations of cholesterol precursors lanosterol and lathosterol in the serum (Figure 2A,E) and was accompanied by a decrease (*p* < 0.01) in lathosterol in the liver (Figure 2F). LE did not significantly affect the parameters of cholesterol biosynthesis except for a decrease of desmosterol concentrations in the liver compared with CON (*p* < 0.05)—LE treatment brought this value back to the level of ICON (Figure 2J).

**Figure 3:**
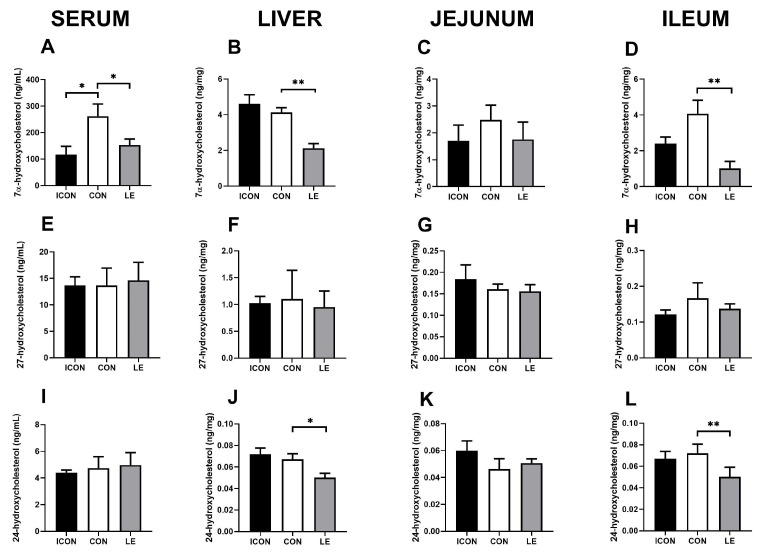
Effects of lemon flavonoid extract Eriomin^®^ (LE) on the concentration of 7α-hydroxycholesterol (**A**–**D**), 27-hydroxycholesterol (**E**–**H**), and 24-hydroxycholesterol (**I**–**L**) in the serum, liver, jejunum, and ileum in old-aged male rats. The groups are abbreviated as follows: intact control (ICON), sunflower oil-treated control (CON), and lemon extract-treated group (LE). Data are expressed as the mean ± SEM (n = 7/group); one-way ANOVA test followed by Dunnet’s post hoc test (CON served as the control group); * *p*< 0.05, ** *p* < 0.01; the remaining comparisons between CON and the study groups were not statistically significant.

Next, we measured the major degradation products of cholesterol (oxysterols: 7α-hydroxycholesterol and 27- and 24-hydroxycholesterol; Figure 3) at the first hydroxylation steps on main (neutral) and alternative (acidic) pathways to bile acids in the serum, liver, jejunum, and ileum. The most striking effect of lemon extract (LE) on cholesterol metabolism was the reduction in 7α-hydroxycholesterol, which is the rate-limiting step in the main pathway of cholesterol degradation to bile acid. This reduction was observed in the serum (*p* < 0.05; Figure 3A), liver (*p* < 0.01; Figure 3B), and ileum (*p* < 0.01; Figure 3D). In addition, the concentration of 24-hydroxycholesterol was significantly lower in the liver and ileum of the LE group compared with concentrations in the CON group (*p* < 0.05; Figure 3J,L), but this reduction was not observed in the serum.

Finally, to investigate the effects of LE on cholesterol absorption, we measured the concentration of plant sterols (campesterol, sitosterol, and stigmasterol) in serum. Plant sterols, structurally similar to cholesterol, can be transported by enterocytes to the lumen of the small intestine but are absorbed at a much lower rate. Therefore, they serve as surrogate markers of cholesterol absorption. Statistical analysis revealed no significant differences among experimental groups. The results are presented in the Supplementary Materials (Figure S1).

### 2.3. Liver Histology, Gene, and Immunohistochemical Expression of CYP7A1 

**Figure 4:**
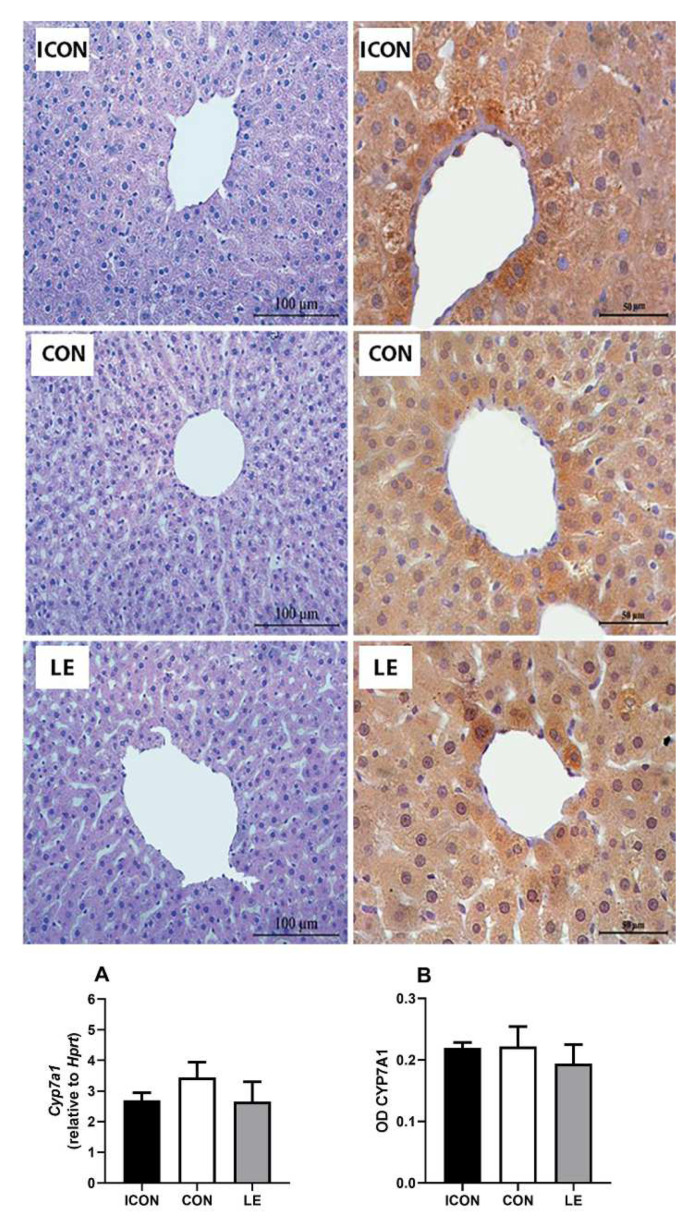
Representative micrographs of hematoxylin and eosin (left panel) and immunohistochemical (right panel) staining showing the localization of CYP7A1 in the liver of intact control (ICON), sunflower oil-treated control (CON), and lemon flavonoid extract Eriomin^®^-treated group (LE) of old-aged male rats; mRNA expression (**A**) and optical density (OD) of CYP7A1 immunostaining in the liver (**B**); data are expressed as the mean ± SEM (n = 7/group); comparisons between the CON and the study groups were not statistically significant (one-way ANOVA test followed by Dunnet’s post hoc test).

The liver histology of old-aged males was characterized by a normal lobular morphology and the usual arrangement of hepatocytes around the central vein. However, hydropic degeneration, manifested as hepatocyte hypertrophy and ballooning, was also observed and was more pronounced in the ICON and CON groups than in the LE-treated old-aged males (Figure 4, left panel). CYP7A1 immunostaining was expressed in a zonal pattern, with maximal expression within a 1–2 cell-thick layer of hepatocytes surrounding the central vein, and the overall expression pattern was maintained after the treatment with LE (Figure 4, right panel). Gene expression (Figure 4A) and CYP7A1 immunostaining intensity (OD; Figure 4B) in the liver were not altered in comparison with the values obtained for CON. 

The data on multivariate tests of significance, including *p*-values, effect sizes, and powers for the gene expression and OD of CYP7A1 immunopositivity are presented in the Supplementary Materials (Tables S1 and S2).

### 2.4. In Silico Analysis

To evaluate the potential capacity of the main active ingredients of lemon extract (eriocitrin, naringin, hesperidin, and didymin) on the metabolism (effect on the main relevant drug-metabolizing biotransformation enzymes of the CYP450 family and stability of the liver microsomal fraction) and different types of toxicity, an interactome analysis was performed using in silico using ADMETlab 3.0. The data obtained are presented in the Supplementary Materials (Tables S3–S6).

We obtained very similar results for all four compounds: all compounds were found to be excellent as inhibitors as well as substrates for CYP1A2, CYP2C19, CYP2C9, CYP2D6, CYP3A4, CYP2B6, and CYP2C8. Human liver microsomal stability (HLM) results were within the normal range, indicating a very low impact on microsomal instability. In terms of potential toxicity, according to the results of our in silico analysis, all ingredients of lemon flavonoids were classified as intermediate in terms of potential hepatotoxicity, mutagenicity, genotoxicity, and ototoxicity. They also have a low risk of carcinogenicity, eye burns and irritation, respiratory damage, nephrotoxicity, neurotoxicity, and immunotoxicity. The value ranges for all substances are given in the Supplementary Materials.

## 3. Discussion

The results of this study indicate that LE primarily acts as an antioxidant against oxidative stress in the liver and small intestine of aged rats. The cholesterol-lowering effect of the lemon extract was not observed in our experimental setup. The decrease in cholesterol degradation products, such as oxysterols 7α-hydroxycholesterol and 24-hydroxycholesterol, as well as the decrease in desmosterol, a direct precursor of cholesterol in its biosynthesis, together with unchanged total cholesterol levels, reflects the maintenance of cholesterol homeostasis. The same oxysterols were decreased in the liver and ileum, highlighting a consistent effect in both organs. Although our results do not confirm our hypothesis regarding the cholesterol-lowering effect of the lemon flavonoid extract Eriomin^®^, the improved pro/antioxidant status in the hepato-intestinal system could be considered very beneficial from a clinical point of view for the elderly population. 

LE had no effect on food intake, weight gain, or liver weight in our study. Naringin and naringenin have been shown to promote weight loss in obesity-induced animal models [23,24]. On the other hand, the potential effects of eriocitrin on weight loss and food intake are not well documented in the literature. Ferreira et al. [25] investigated the effect of lemon extract at different doses (25, 50, and 100 mg/kg b.w.) during a four-week treatment and found that only the treatment with 100 mg/kg of eriocitrin increased food intake and weight gain. In addition, Fukuchi et al. [26] showed that dietary supplementation with 0.5% (*w*/*w*) lemon extract for 12 weeks in mice fed a high-fat or low-fat diet reduced body weight only in the animals fed a high-fat diet.

Polyphenols’ limited water solubility and low bioavailability are limiting factors for their practical usage. An interesting finding from this investigation is that sunflower oil per se has a tissue-specific effect on the parameters of oxidative stress. It has no significant influence on the liver but promotes a pro-oxidant status in the jejunum while enhancing antioxidant status in the ileum. However, it led to a reduction in the concentrations of cholesterol precursors, especially lanosterol in serum and lathosterol in both serum and liver. Sunflower oil contains low concentrations of saturated fats and high amounts of polyunsaturated fats (PUFAs) and monounsaturated fatty acids as well as vitamin E [27,28]. PUFAs contain double bonds, and they are highly susceptible to oxidative stress. Consequently, the oxidation of PUFAs and the resulting increase in lipid peroxidation (LPO) occur [29], which is consistent with our data obtained for oxidative stress parameters in the jejunum of the CON group. The aged jejunum is characterized by lower antioxidant defenses compared with young adults [9]. Moreover, PUFAs and vitamin E have been reported to inhibit cholesterol metabolism and biosynthesis [30,31]. 

In our study, there were no changes in the activity of antioxidant defense enzymes and the concentrations of oxidative damage parameters (except for a lowering of LPO in the jejunum) between the LE and CON groups of animals. However, if the differences are expressed as a percentage, CAT activity in the liver of the LE group increased by 17.9%, GSH-Px activity by 19.0%, and GR activity by 43.1% compared with the CON group. These increases were not significant but could indirectly indicate increased expression of Nrf2 and other redox regulators as well as somewhat increased gene and/or protein expression of glutathione peroxidase and reductase, as previously demonstrated in the liver of LE-treated aged rats [7]. Moreover, our findings on the lower activities of SOD and CAT in the aged rat liver [9] are in line with data from other researchers, indicating that this tissue is more susceptible to oxidative stress during aging [32]. In addition, oxidative stress is known to contribute to the age-related increase in the spectrum of non-alcoholic fatty liver disease [33]. The TOS results in the LE group suggest a reduced pro-oxidant effect during aging, possibly as an adaptive response to long-term exposure to ROS during the aging process. However, TAS was also reduced in the liver of the LE group compared with the CON group but to a smaller extent than TOS, thus probably leading to improved OSI. TAS primarily measures the antioxidant protective capacity concerning non-enzymatic, low-molecular weight components of the antioxidant system (AOS). It can be concluded that the LE treatment led to a reduction of these low-molecular weight components, possibly by preserving them through its antioxidant effect and scavenging of ROS. Consistent with this, the administration of citrus flavonoid naringenin to middle-aged ApoE^−^/^−^ mice suppressed hepatic steatosis and reduced hepatic oxidative stress by inhibiting ROS generation and normalizing the activities of antioxidant enzymes [34].

The oxidative stress index (OSI) shows the deviation from the normal state of oxidative balance (zero value), i.e., the perfect balance between the pro- and antioxidant components of oxidative equilibrium. Low values of the OSI index reflect an oxidation state that is closer to oxidative equilibrium. The OSI index increases proportionally with each degree of oxidative imbalance; its increase can be caused either by an increase in the pro-oxidant component or by a decrease in antioxidant protection. An analysis of all oxidative stress parameters indicates that treatment with LE lowered the OSI consistently in all examined organs of 24-month-old rats by quenching ROS. This property of flavonoids from lemon extract is well documented in the literature [35,36] and is confirmed in the present study.

In the liver, only desmosterol, the immediate precursor of cholesterol in the Bloch pathway, is significantly reduced in the LE group in comparison with concentrations obtained for CON. Our results show that LE only affected the Bloch pathway and not the Kandutsch–Russell pathway of cholesterol biosynthesis, in which the main intermediate compound is lathosterol [37]. Concentrations of an early precursor of cholesterol biosynthesis, lanosterol, were also somewhat reduced, though not significantly, indicating a small interaction of LE at an earlier, maybe HMG-CoA level. Further studies with probably higher concentrations of LE and a prolonged treatment might confirm this “statin-like” possibility. Our results indicate that hepatic desmosterol, as an immediate precursor of cholesterol, served as an alternative substrate for cytochrome P450 enzymes involved in the degradation of cholesterol to bile acids, including CYP7A1 and CYP46A1 [38,39], which might explain reduced desmosterol concentrations under our experimental conditions.

Oxysterols, known as cholesterol oxidation products, have gained attention as diagnostic biomarkers for oxidative stress, as intermediates in cholesterol degradation for bile acid synthesis, and as messengers in cell signaling [40]. 7α-, 27-, and 24-hydroxycholesterol were measured in the serum and liver. In the serum and liver, 7α-hydroxycholesterol concentrations were reduced, which is expected, as serum 7α-hydroxycholesterol is solely produced by the liver, by the activity of CYP7A1 in the main pathway of bile acid synthesis, or non-enzymatically by autoxidation from the oxidation of free radical chains [41]. 27-hydroxycholesterol is also formed in the liver in an alternative pathway of bile acid formation, but this pathway is much less activated in healthy tissue [42]. On the other hand, the majority of circulating 24-hydroxycholesterol is produced in the brain, but in murine species, around 50% is also produced peripherally (CYP46A1) in the liver [43]. Therefore, the reduction of 24-hydroxycholesterol contents by LE indicates that this effect is hepatic only. Taken together, our data, including reduced 7α-hydroxycholesterol concentrations in both the serum and liver, indicate that bile acid synthesis is potentially reduced, which may be interpreted as a negative effect of LE. However, as this was counterbalanced by reduced hepatic cholesterol synthesis, leading to unchanged cholesterol concentrations, it is neither positive nor negative. However, this finding is important, particularly considering the well-established interactions of grapefruit flavonoids with certain CYP enzymes, which can affect the bioavailability of some statin drugs [44] widely used among the aged population. The results of our in silico analysis indicate that all main active ingredients of lemon extract (eriocitrin, naringin, hesperidin, and didymin) do not interact with the respective CYP450 drug-metabolizing enzymes as inhibitors or substrates. Moreover, a local decrease in 7α- and 24-hydroxycholesterol was also evident in the ileum of LE group, clearly indicating the consistent effect of LE. The obtained results might indicate a change in bile acid composition and/or enhanced bile absorption in the distal ileum upon LE treatment. The measurement of bile acid synthesis in future studies by fecal excretion rates is necessary to confirm the hypothesis that bile acid absorption is enhanced. 

Histopathological analysis revealed no hepatotoxicity of examined dose of LE, despite in silico predictions of medium hepatotoxicity for its major ingredients. About 70% of toxicity-related events are evidenced preclinically (in cells and animals; https://admetlab3.scbdd.com/explanation/#/, accessed on 1 April 2024). Moreover, LE did not affect hepatic gene or IHC expression of CYP7A1 in the liver and ileum. Therefore, it is reasonable to speculate that the activity of these cytochrome P450 enzymes is inhibited at the post-transcriptional level, possibly due to the apparent free radical scavenging ability of the lemon flavonoid extract. The same mechanism may contribute to the lower levels of 24-hydroxycholesterol and desmosterol. Sterols from cholesterol synthesis were reported to undergo further oxidation to metabolites that are not involved in cholesterol production by the activity of CYP enzymes [38]. Further studies are needed to confirm this assumption. 

## 4. Materials and Methods

### 4.1. Animal Experiments and Study Design 

The male Wistar rats used in this experiment were bred and housed in the Unit of Experimental Animals of the Institute for Biological Research “Siniša Stanković”—National Institute of the Republic of Serbia, Belgrade, Serbia. The rats were kept in a controlled environment with a 12 h light–dark cycle and a constant temperature of 21 ± 2 °C. They had ad libitum access to a normal grain-based pellet diet (IG-Z-00117, Gebi d.o.o., Čantavir, Serbia) and water. All procedures involving the animals were complied with Directive 2010/63/EU on the protection of animals used for scientific purposes and were approved by the Ethics Committee for the Use of Laboratory Animals of IBISS, University of Belgrade (approval number 2-12/12). The experiments were performed following the guidelines of ARRIVE (Animal Research: Reporting In Vivo Experiments) and the guidelines on the principles of regulatory acceptance of 3R (replacement, reduction, refinement) testing approaches. 

Twenty-four months old rats were randomly divided into three experimental groups, consisting of seven animals per group. Animals from the LE group were orally provided a daily dose of 40 mg/kg body weight of lemon flavonoid extract Eriomin^®^ (Nature TM, Montclair, CA, USA) suspension mixture in sunflower oil (volume of 0.3 mL) for 4 weeks. The suspension mixture was directly injected into the oral cavity. The control group (CON) received the same volume of sunflower oil alone during the same period, while the control group with intact age (24 months old; ICON) received no treatment. Eriomin^®^ is a semi-purified mixture of citrus flavonoids extracted from commercial sources of lemon (*Citrus limon* (L.) Burm. f.) juice and peel, with a standardized composition containing 70% eriocitrin, 5% hesperidin, 4% naringin, and 1% didymin, as specified by the manufacturer and determined by high-performance liquid chromatography. Additionally, the extract contained 20% fiber material, comprising suberin, cutin, lignin, pectin, and cellulose [12].

The selected dose of 40 mg/kg body weight according to allometric calculation [44] corresponds to a nutritive relevant dose of citrus flavonoids (almost 300 mg/day) in humans [45]. Direct administration into the oral cavity was chosen because the oral microbiota plays an important role in the initial metabolism of polyphenols and the release of metabolites, which are considered to be more biologically active and better absorbed than the original polyphenolic compounds [46]. LE was poorly soluble in water, and an aqueous suspension had a bitter taste and low viscosity, necessitating administration by gavage. Therefore, we chose edible oil (which is widely used in the human diet) as a non-toxic, non-bactericidal, neutral, and viscous carrier in which we suspended the lemon flavonoid extract. Sunflower oil was tasty and palatable to rodents, as it mimicked the bitter taste of the lemon extract, and all these properties were crucial for ensuring adequate control over the delivery of the selected dose in the oral cavity. Sunflower oil contains fewer polyphenols compared with other dietary oils, which was important for reducing interference with the results we expected from the polyphenols in the lemon extract [47].

### 4.2. Sample Collection 

Twenty-four hours after the last treatment, the animals were decapitated without anesthesia. After decapitation, the whole blood was collected from the trunk. After the blood was allowed to clot, the serum was separated by centrifugation at 3000× *g* for 15 min and stored at −80 °C. The liver was removed, and the wet weight was measured. The relative liver weight was calculated for each animal as a percentage of the absolute liver weight with the total body mass. The samples of proximal jejunum and distal ileum were promptly rinsed with a cold saline solution. The tissue samples of liver and small intestine were either further processed for tissue histology or stored after shock-freezing in liquid nitrogen at −80 °C for other analyses. 

### 4.3. Quantification of Oxidative Stress Parameters in the Liver, Jejunum, and Ileum

To determine oxidative stress parameters, the liver, jejunum, or ileum samples were minced and homogenized in 10 volumes of a solution containing 25 mmol/L sucrose and 10 mmol/L Tris-HCl at pH 7.5. This solution was supplemented with 1× phosphatase inhibitor mix I and 1× protease inhibitor mix G. Homogenization was carried out at 1500 rpm using an IKA-Werk Ultra-Turrax homogenizer from Janke and Kunkel (Staufen, Germany) at 4 °C. Homogenates were then sonicated on ice at 10 kHz for 30 s using a Bandeline Sonopuls HD 2070, followed by centrifugation in a Beckman ultracentrifuge at 100,000× *g* for 90 min at 4 °C [48]. The resulting supernatants were used for biochemical analyses. The activity of antioxidant defense enzymes was measured for each sample in triplicate using a Shimadzu UV-1900i spectrophotometer (Shimadzu Corporation, Nishinokyo-Kuwabaracho, Nakagyo-ku, Kyoto, Japan) with a temperature-controlled cuvette holder. The activity of superoxide dismutase (SOD, EC 1.15.1.1) was determined using the epinephrine method [49] and expressed as U/g wet mass. Catalase (CAT) activity (EC 1.11.1.6) was assessed by the rate of hydrogen peroxide (H_2_O_2_) degradation and expressed as µmol H_2_O_2_/min/g wet mass [50]. The activity of glutathione peroxidase (GSH-Px, EC 1.11.1.9) was determined by following the oxidation of nicotinamide adenine dinucleotide phosphate (NADPH), which served as a substrate, with t-butyl hydroperoxide [51] and was expressed as nmol NADPH/min/g wet mass. Glutathione reductase (GR, EC 1.8.1.7) activity was measured according to the method of Habig et al. [52], which is based on the ability of GR to catalyze the reduction of oxidized glutathione GSSG) to reduce glutathione (GSH) with NADPH as a substrate in phosphate buffer (pH 7.4). GR activity was expressed as nmol NADPH/min/mg protein. The activity of glutathione S-transferase (GST, EC 2.5.1.18) towards 1-chloro-2,4-dinitrobenzene (CDNB) was determined according to the method of Glatzle et al. [53] and expressed as nmol GSH/min/g wet mass. The concentration of total GSH was determined as described by Griffith [54] and expressed as nmol/g wet mass. Concentrations of SH groups were determined using DTNB according to the method described by Ellman [55] and expressed as µmol/g wet mass. The concentration of lipid peroxidation products, measured as reactive substances of thiobarbituric acid (TBARS) in animal tissue, was determined according to the method of Rehncrona et al. [56] using thiobarbituric acid (TBA) and expressed as nmol TBARS/g wet mass. The protein content (PCO carbonylation) was determined according to the method of Mesquita et al. [57] based on the reaction of 2,4-dinitrophenylhydrazine (DNPH) with carbonyl groups (C=O) to form 2,4-dinitrophenylhydrazone and expressed as (nmol/g wet mass).

Total oxidant status (TOS) and total antioxidant status (TAS) were determined using colorimetric assay kits (Elabscience, Houston, TX, USA) according to the manufacturer’s recommendations. The principle of TOS determination is that the oxidizing material in the sample can oxidize Fe^2+^ to Fe^3+^ under acidic conditions, which combines strongly with xylenol orange to form a blue-purple complex. The maximum absorption wavelength is given at 590 nm at pH 2–3. TOS was expressed as μmol H_2_O_2_ equiv./g wet mass. The detection principle of TAS determination is based on the oxidation of ABTS, 2,2′-azino-bis (3ethylbenzothiazoline-6-sulfonic acid), by a suitable oxidizing agent to green ABTS-+, which can be reduced to colorless ABTS in the presence of antioxidants. The TAS of the sample was determined at 660 nm. Trolox was used as a reference substance for total antioxidant status. TAS was expressed as mmol Trolox equiv./g wet mass. Both TOS and TAS were measured using a TAC-SYNERGY H1 microplate reader (BioTek, Agilent, Santa Clara, CA, USA). OSI was calculated as the ratio of (TOS/TAS) × 100. The OSI was expressed in arbitrary units (AUs) [58].

### 4.4. Quantification of Sterols and Oxysterols in the Serum, Liver, Jejunum, and Ileum

Serum samples (100 µL) were directly used to measure sterol and oxysterol concentrations. Liver, jejunum, and ileum samples were aliquoted and dried in a Speedvac concentrator (Savant DNA120, Thermo Scientific GmbH, Karlsruhe, Germany) at room temperature. The dry weight of these samples was used as the basis for calculating the concentrations of sterols and oxysterols. Chloroform/Methanol (2:1) was used for the extraction of sterols and oxysterols, and 100 µL of the resulting sterol extract was used for further processing. A total of 50 µg of 5α-cholestane (Serva, Heidelberg, Germany) (50 μL from a stock solution of 5α-cholestane in cyclohexane [Merck KGaA, Darmstadt, Germany]; 1 mg/mL) was added as an internal standard (ISTD) for cholesterol quantification by gas chromatography–flame ionization detection, 1 μg of epicoprostanol (Sigma, Deisenhofen, Germany) (10 μL from a stock solution of epicoprostanol in cyclohexane; 100 μg/mL) was added as an ISTD for the quantification of the non-cholesterol sterols (cholesterol precursors: lanosterol, desmosterol, and lathosterol), and 100 ng of deuterium-labeled oxysterols [26.26.26.27.27.27-2H6]7α-hydroxycholesterol, [2.2.3.4.6-2H5]27-hydroxycholesterol, and [22.23.24.25-2H4]24-hydroxycholesterol (Medical Isotopes, Pelham, NH, USA) were added as an ISTD for the respective authentic oxysterols (cholesterol metabolites: 7α-, 27-, and 24-hydroxycholesterol).

After saponification with 2 mL of 1M 95% ethanolic sodium hydroxide solution (Merck KGaA, Darmstadt, Germany) at 60 °C for one hour, the free sterols were extracted three times with 3 mL of cyclohexane. The organic solvent was evaporated by a gentle stream of nitrogen at 60 °C on a heating block. The residue was dissolved in 80 µL of n-decane (Merck KGaA, Darmstadt, Germany). An aliquot of 40 µL was incubated (1 h at 70 °C on a heating block) with the addition of 20 µL of trimethylsilylating (TMSi) reagent (chlortrimethylsilane [Merck KGaA, Darmstadt, Germany]/1.1.1.3.3.3-Hexamethyldisilasane [Sigma Aldrich, Co., St. Louis, MO, USA]/pyridine [Merck KGaA, Darmstadt, Germany], 9:3:1) in a GC vial for gas chromatography–mass spectrometry–selected ion monitoring (GC–MS–SIM) non-cholesterol analysis. Another aliquot of 40 µL was incubated by the addition of 40 µL of the TMSi-reagent and dilution with 300 µL of n-decane in a GC vial for GC-FID cholesterol analysis.

Gas chromatographic separation and detection of cholesterol and 5a-cholestane (ISTD) was performed on a DB-XLB 30 m × 0.25 mm i.d. × 0.25 µm film thickness (J&W Scientific Alltech, Folsom, CA, USA) in a Hewlett-Packard (HP) 6890 Series GC-system (Agilent Technologies, Palo Alto, CA, USA) equipped with a flame ionization detector (FID).

The silylated non-cholesterol sterols (epicoprostanol, ISTD) and the disilylated oxysterols (2Hx-oxysterols, ISTD) were separated on another DB-XLB column (30 m × 0.25 mm i.d. × 0.25 µm film thickness, J&W Scientific Alltech, Folsom, CA, USA) in an HP 6890N Network GC system (Agilent Technologies, Waldbronn, Germany) connected with a direct capillary inlet system to a quadrupole mass-selective detector HP5975B inert MSD (Agilent Technologies, Waldbronn, Germany). Both GC systems were equipped with HP 7687 series autosamplers and HP 7683 series injectors (Agilent Technologies, Waldbronn, Germany).

An aliquot of 2 µL was injected by automated injection in a splitless mode using helium (1 mL/min) as a carrier gas for GC–MS–SIM and hydrogen (1 mL/min) for GC-FID analysis at an injection temperature of 280 °C. The temperature program for both GCs was as follows: 150 °C for three min, followed by 20 °C/min for up to 290 °C for 34 min. For MSD, electron impact ionization was applied with 70 eV. SIM was performed by cycling the quadrupole mass filter between different m/z at a rate of 3.7 cycles/s. Non-cholesterol sterols were monitored as their TMSi- and the oxysterols as their di-TMSi-derivatives using the following masses: epicoprostanol m/z 370, lathosterol at m/z 458, desmosterol at m/z 441, lanosterol at m/z 393, [26.26.26.27.27.27-2H6]7α-hydroxycholesterol at m/z 462, [2.2.3.4.6-2H5]27-hydroxycholesterol at m/z 461, [22.23.24-2H3]24-hydroxycholesterol at m/z 416, 7α-hydroxycholesterol at m/z 456, 24-hydroxycholesterol at m/z 413, and 27-hydroxycholesterol at m/z 456. Peak integration was performed manually. Cholesterol was directly quantified by multiplying the ratios of the area under the curve of cholesterol to 5α-cholestane by 50 µg (ISTD amount). Non-cholesterol sterols and oxysterols were quantified from the ratios of the areas under the curve of the respective non-cholesterol sterols or oxysterols after SIM analyses against epicoprostanol or deuterium-labeled oxysterols, respectively, using standard curves for the listed sterols/oxysterols. The identity of all sterols and oxysterols was established by a comparison with the full-scan mass spectra of authentic compounds. Additional qualifiers (characteristic fragment ions) were used for structural identification (m/z values not shown).

### 4.5. Gene Expression Analyses of Cyp7a1 in the Liver

Total RNA from livers was isolated using TRIzol (Invitrogen, Carlsbad, CA, USA) according to the manufacturer’s instructions. A volume equivalent to 1 µg of RNA was used for reverse transcription to generate cDNA using the High-Capacity cDNA Reverse Transcription Kit (Applied Biosystems, Vilnius, Lithuania). Quantitative real-time PCR (qRT-PCR) was performed to assess the mRNA expression level in a real-time PCR machine ABI Prism 7000 (Applied Biosystems, Waltham, MA, USA) with SYBRGreen PCR Master Mix (Applied Biosystems, USA), exactly as previously reported. The sequences of the oligonucleotides designed with PrimerBLAST (NCBI) were: *Cyp7a1*-f: 5′-CACCATTC CTGCAACCTTTT-3′; *Cyp7a1*-r: 5′-GTACCGGCAGGTCATTCAGT-3′; Hprt–f: 5′-TATGGACAGGACTGAAAGACTTG-3′; and Hprt–r: 5′-CAGCAGGTCAGCAAAGAACTTATA-3′. Gene expression was calculated by the delta–delta CT method, using Hprt as an endogenous control for normalization.

### 4.6. Liver Histology and Immunohistochemical Analysis of CYP71

Liver samples were taken and fixed in Bouin’s fixative for 48 h. After fixation, the tissues were dehydrated in a series of ascending ethanol concentrations (30–100%) and then embedded in Histowax^®^ (Histolab Product AB, Gothenburg, Sweden). Sections of liver that were 5 µm thick were stained with hematoxylin and eosin (HE) or immunostained with rabbit antiserum directed against CYP7A1 (1:100; Abcam, Cambridge, UK; overnight at 4 °C), exactly as previously reported [9]. For immunodetection, the VECTASTAIN^®^ ABC (rabbit IgG; Vector Laboratories, Newark, CA, USA) kit was used with the biotin/avidin system according to the recommended procedure. All washes and dilutions were performed with 0.1 mol/L PBS, pH 7.2. Hematoxylin was used as a counterstain, and slides were then mounted in DPX medium (Sigma-Aldrich, Barcelona, Spain). 

All digital images were acquired using a LEITZ DM RB photomicroscope (Leica Microscopy and Systems GmbH, Wetzlar, Germany), with a Leica DFC 320 CCD camera (Leica Microsystems Ltd., Heerbrugg, Switzerland) and Leica DFC Twain software version 7.0 (Leica, Germany) used for image acquisition and analysis.

Quantification of the DAB IHC signal in the liver hepatocytes was performed using the Windows-based software ImageJ Fiji (Image J, version 1.49j) with the open-source plugin IHC profiler, as we have previously described in detail [9]. The optical density (OD) was calculated as OD = log (maximum intensity/mean intensity). For the analysis, six randomly acquired images (2088 × 1550 pixels × 40 objective magnification) per liver tissue per animal were analyzed.

### 4.7. In Silico Analysis

The potential effects of main components of lemon flavonoid extract (eriocitrin, hesperidin, naringin, and didymin) on CYP450 enzymes-mediated metabolism (CYP1A2, CYP2C19, CYP2C9, CYP2D6, CYP3A4, CYP2B6 and CYP2C8) and liver microsomal stability, as well as potential toxicity of the main components, were evaluated using ADMETlab 3.0 for in silico analysis (Computational Biology and Drug Design Group © 2023).

### 4.8. Statistics

Data were analyzed using the statistical programs GraphPad Prism for Windows (v.8, San Diego, CA, USA) and STATISTICA software (v. 12.5, Palo Alto, CA, USA). The normality of the distribution of the data was checked with the Shapiro–Wilk and Kolmogorov–Smirnov tests (n < 50) [59]. A one-way ANOVA analysis followed by a Dunnett’s post-hoc test was used, in which the effects of both ICON and LE groups were compared with the values of the selected control, vehicle-treated, and CON group. Multivariate tests of significance, effect sizes, and powers, as well as probabilities for post-hoc tests (2-sided) were performed. Differences were considered statistically significant at * *p* < 0.05, ** *p* < 0.01, *** *p* < 0.001, and **** *p* < 0.0001, with 95% confidence intervals. All results are expressed as the mean ± standard error of the mean (SEM).

## 5. Conclusions

In summary, our study demonstrates that lemon extract primarily acts as an antioxidant in the livers and small intestine of aged rats, with no significant cholesterol-lowering effect observed. The antioxidant properties of the extract may influence the activity of CYP7A1 and other enzymes involved in cholesterol degradation to bile acids at the post-transcriptional level. Our data show a consistent reduction in 7α-hydroxycholesterol concentrations in the serum, liver, and ileum, as well as a reduction in hepatic and ileal 24-hydroxycholesterol levels, suggesting a potential decrease in cholesterol degradation to bile acids following lemon extract treatment. However, this decrease is counterbalanced by the decrease in cholesterol biosynthesis, as evidenced by lower hepatic desmosterol concentrations, resulting in unchanged total cholesterol levels. Further studies are recommended to explore the potential effects of LE on bile acid metabolism. Specifically, investigating serum bile acid concentrations and conducting fecal balance analyses using markers for cholesterol and bile acid excretion and absorption could elucidate these effects. Furthermore, the limited solubility of lemon flavonoid extract opens up the possibility of developing specialized delivery systems that could enhance its biological effects.

## Figures and Tables

**Table 1:** Effect of lemon extract on body weight gain, food intake, and liver weight in old-aged male rats.

	ICON	CON	LE	*p*-Value (CON vs. ICON)	*p*-Value (CON vs. LE)
Initial body weight (g)	450 ± 16	467 ± 8	456 ± 9	0.902	0.950
Weight gain (g)	−12.0 ± 3	−16.9 ± 2	−15.0 ± 2	0.841	0.987
Food intake (g)	17.8 ± 1.0	18.1 ± 1.1	17.5 ± 1.2	0.834	0.739
Absolute liver weight (g)	13 ± 1	11 ± 1	12 ± 1	0.241	0.676
Relative liver weight (% b.m.)	2.7 ± 0.1	2.4 ± 0.1	2.5 ± 0.1	0.147	0.601

Results are the mean ± SEM (n = 7/group). The groups are abbreviated as intact control (ICON), sunflower oil-treated control (CON), and lemon extract-treated (LE); one-way ANOVA test followed by Dunnet’s post hoc test (CON served as the main control group).

**Table 2:** Activities of superoxide dismutase (SOD, U/g w.m.), catalase (CAT, U/g w.m.), glutathione peroxidase (GSH-Px, U/g w.m.), glutathione reductase (GR, U/g w.m.), and glutathione S-transferase/GST, U/g w.m.) and the concentrations of total glutathione (GSH, nmol/g w.m.), sulfhydryl groups (SH, μmol/g w.m.), lipid peroxidation products (LPO, nmol/mg w.m.), protein carbonyls (PCO, μmol/g w.m.), total oxidant status (TOS, µmol H_2_O_2_/L), total antioxidant status (TAS, µmol Trolox/L), and oxidative stress index (OSI, AU) in the liver, jejunum, and ileum of old-aged male rats.

LIVER			
	ICON	CON	LE
SOD	3349.8 ± 68.6	3300.5 ± 230.5	3543.7 ± 196.6
CAT	52,352.5 ± 6942.4	48,246.4 ± 8190.7	56,875.0 ± 4214.8
GSH-Px	142,292.6 ± 21,438.2	121,866.5 ± 18,844.4	144,966.5 ± 15,314.2
GR	6857.3 ± 401.4	3766.9 ± 1486.9	5390.3 ± 1330.5
GST	173,576.6 ± 10,808.7	166,185.9 ± 26,910.3	169,963.8 ± 11,768.9
GSH	3854.4 ± 235.0 *	5959.1 ± 203.4	5664.7 ± 406.0
SH	3681.8 ± 315.6 *	4600.6 ± 147.4	4674.4 ± 211.6
LPO	11.9 ± 1.4	11.9 ± 1.2	13.6 ± 0.8
PCO	3340.7 ± 161.2	4271.4 ± 420.0	3653.8 ± 266.6
TOS	34.4 ± 0.6	37.8 ± 2.5	14.1 ± 0.6 ****
TAS	873.3 ± 95.0	1002.1 ± 105.1	706.2 ± 56.9 ***
OSI	3.96 ± 0.37	3.90 ± 0.55	2.03 ± 0.11 ***
**JEJUNUM**			
SOD	942.5 ± 99.1	1585.4 ± 222.6	1384.1 ± 349.6
CAT	302.6 ± 81.2	338.4 ± 46.4	347.6 ± 89.1
GSH-Px	2416.4 ± 532.8	3668.0 ± 492.5	3689.7 ± 370.1
GR	3376.2 ± 515.0	3885.0 ± 285.9	3569.1 ± 726.8
GST	11,935.4 ± 759.6	16,866.7 ± 1540.4	13,458.3 ± 5636.5
GSH	615.8 ± 44.5 *	425 ± 63.0	425.8 ± 55.3
SH	1048.1 ± 149.4	1108.5 ± 192.9	1325.1 ± 308.3
LPO	8.8 ± 1.5 **	2.3 ± 0.6	1.6 ± 0.6 **
PCO	1304.5 ± 205.8	2093.4 ± 647.0	1869.3 ± 335.9
TOS	29.9 ± 1.8 *	35.7 ± 1.4	16.9 ± 1.9 ***
TAS	1695.0 ± 127.4 *	1333.3 ± 32.8	2103.3 ± 49.1 *
OSI	1.16 ± 0.47 *	2.86 ± 0.20	0.66 ± 0.06 ***
**ILEUM**			
SOD	566.5 ± 17.4	715.8 ± 77.6	721.2 ± 36.0
CAT	142.6 ± 40.0	127.6 ± 21.6	120.9 ± 17.3
GSH-Px	2866.6 ± 167.9	2483.9 ± 192.3	2681.7 ± 273.2
GR	4420.4 ± 636.3	5020.9 ± 295.7	4336.0 ± 620.6
GST	5512.5 ± 507.2	4422.9 ± 760.3	3995.8 ± 226.0
GSH	295.0 ± 6.2 *	420.0 ± 71.7	404.6 ± 45.9
SH	1034.6 ± 115.9	1000.7 ± 39.1	883.7 ± 103.6
LPO	5.1 ± 0.4	5.2 ± 0.4	7.5 ± 1.4
PCO	1609.3 ± 349.5	1557.7 ± 284.4	1490.5 ± 341.4
TOS	32.2 ± 3.3	37.6 ± 0.7	29.9 ± 1.6 *
TAS	1760.0 ± 70.7 ***	1030.0 ± 13.3	1856.7 ± 123.5 **
OSI	1.872 ± 0.26 **	3.653 ± 0.02	1.04 ± 0.31 ***

Data are presented as the mean ± SEM (n = 7/group). The groups are abbreviated as intact control (ICON), sunflower oil-treated control (CON), and lemon extract-treated (LE); one-way ANOVA test followed by Dunnet’s post hoc test (CON served as the main control group; * *p* < 0.05; ** *p* < 0.01; *** *p* < 0.001; **** *p* < 0.001; the remaining comparisons between CON and the study groups were not statistically significant).

## Data Availability

The data supporting the conclusions of this article will be made available by the authors upon reasonable request.

## Supplementary Materials

The following supporting information can be downloaded at: https://www.mdpi.com/article/10.3390/ijms25105221/s1.
