# Supplementary material for: Lemon Flavonoid Extract Eriomin Improves Pro/Antioxidant Status and Interferes with Cholesterol Metabolism without Affecting Serum Cholesterol Levels in Aged Rats
Source: Int J Mol Sci. 2024 May 10;25(10):5221. doi: 10.3390/ijms25105221 (PMC11121178; doi:10.3390/ijms25105221)
Supplement: Supplementary file 1 [file ijms-25-05221-s001.zip › ijms-2966634-supplementary.pdf]

Supplementary Materials

Table S1. Multivariate tests of significance, effect sizes, and powers. Sigma-restricted Parameterization Effective Hypothesis Decomposition

Effect	Test	Value	F	Effect df	Error df	p-value	Partial eta-squared	Non-centrality	Observed power (α=0.05)
**Effect of group**									
Intercept	Wilks	0.000843	197.6399	6	1	0.054395	0.999157	1185.840	0.642775
Groups	Wilks	0.280371	0.1481	12	2	0.989152	0.470499	1.777	0.057159
**Effect of tissue**									
Intercept	Wilks	0.003610	261.5143	19	18	0.000000	0.996390	4968.772	1.000000
Tissue	Wilks	0.001315	25.1791	38	36	0.000000	0.963739	956.806	1.000000

Table S2. Results of One-way ANOVA of the comparison between groups for the investigated parameters.

		**LIVER**		
**Variable**	**MS**	**F**	**p-value**	**SEM**
**SOD**	79641	0.4028	0.677940	197738
**CAT**	1.193864E+08	0.5867	0.572646	2.034806E+08
**GSH-Px**	8.822080E+08	0.6635	0.534505	1.329668E+09
**GR**	7109209	1.21233	0.334343	5864090
**GST**	2.305335E+07	0.0177	0.982476	1.301846E+09
**GSH**	4924593	10.3319	0.002982 *	476640
**SH**	4.190	0.8682	0.001419 *	4.826
**LPO**	2228435	12.620	0.141900	176579
**PCO**	745045	1.5974	0.246010	466415
**TOS**	742.19	95.556	0.000000 *	7.77
**TAS**	121976	5.7426	0.019598 *	21.240
**OSI**	5.3364	13.1717	0.001204 *	0.4051
**Total Cholesterol**	1.802	2.105	0.149379	0.856
**Lanosterol**	7.9465	1.65852	0.216827	4.7913
**Lathosterol**	0.33890	3.6585	0.045280 *	0.09263
**Desmosterol**	0.000070	17.7191	0.000045 *	0.000004
**7α-hydroxycholesterol**	12.4056	13.2166	0.000253 *	0.9386
**27-hydroxycholesterol**	0.48105	1.06702	0.363768	0.45083
**24-hydroxycholesterol**	0.000929	4.9881	0.018145 *	0.000186
**Cyp7a1 gene expression**	0.9163	0.38038	0.693084	2.4089
**OD CYP7A1**	0.000139	0.0749	0.928350	0.001858
		**SERUM**		
**Total Cholesterol**	1.802	2.105	0.149379	0.856
**Lanosterol**	0.33890	3.6585	0.045280 *	0.09263
**Lathosterol**	0.000070	17.7191	0.000045 *	0.000004
**Desmosterol**	7.9465	1.65852	0.216827	4.7913
**7α-hydroxycholesterol**	0.000929	4.9881	0.018145 *	0.000186
**27-hydroxycholesterol**	0.48105	1.06702	0.363768	0.45083
**24-hydroxycholesterol**	0.020205	2.7824	0.109280	0.340904
		**JEJUNUM**		
**SOD**	637296	3.0393	0.085528	209688
**CAT**	2796	0.10824	0.89828	25828
**GSH-Px**	2852901	2.0605	0.170117	1384592
**GR**	392919	0.3472	0.713548	1131749
**GST**	3.754228E+07	1.6140	0.239450	2.325975E+07
**GSH**	64879	3.9503	0.048071 *	16424
**SH**	78581	0.40006	0.678896	196422
**LPO**	79.0952	13.39477	0.000877 *	5.9049
**PCO**	968495	0.80114	0.471428 *	1208891
**TOS**	350.64	23.0321	0.000078 *	15.22
**TAS**	612745	13.7760	0.000780 *	44479
**OSI**	6.51393	9.88888	0.002900 *	0.65871
**Total Cholesterol**	1.8769	0.5211	0.606710	3.6018
**Lanosterol**	2.26315	2.11206	0.163727	1.07154
**Lathosterol**	0.003279	1.58265	0.245459	0.002072
**Desmosterol**	14378.1	1.258062	0.319142	11428.7
**7α-hydroxycholesterol**	1.45518	0.77205	0.483710	1.88482
**27-hydroxycholesterol**	0.000750	0.1960	0.824606	0.003828
**24-hydroxycholesterol**	0.000520	2.0283	0.174250	0.000256
		**ILEUM**		
**SOD**	39500	3.0548	0.088071	12930
**CAT**	2844.4	0.69528	0.519589	4091.0
**GSH-Px**	146533	0.7327	0.502640	199977
**GR**	385768	0.2933	0.751434	1315151

**GST**	3763426	2.3901	0.137417	1574588
**GSH**	44784	3.8247	0.045828 *	11709
**SH**	20152	0.5177	0.609718	38927
**LPO**	3.1074	1.1571	0.349892	2.6855
**PCO**	128310	0.25854	0.776739	496279
**TOS**	91.28	3.8314	0.045612 *	23.82
**TAS**	965308	37.073	0.000013 *	26038
**OSI**	780.018	33.6690	0.000020 *	23.16
**Total Cholesterol**	5.455	2.0678	0.157100	2.638
**Lanosterol**	0.2456	0.17221	0.843250	1.4263
**Lathosterol**	0.002207	0.862337	0.439840	0.002559
**Desmosterol**	27106	0.48513	0.623886	55875
**7α-hydroxycholesterol**	14.5237	6.19055	0.009555 *	2.3461
**27-hydroxycholesterol**	0.003650	2.2053	0.140753	0.001655
**24-hydroxycholesterol**	0.000831	9.3917	0.001789 *	0.000089

Significantly differences between groups after One-way ANOVA: *$p<0.05$.

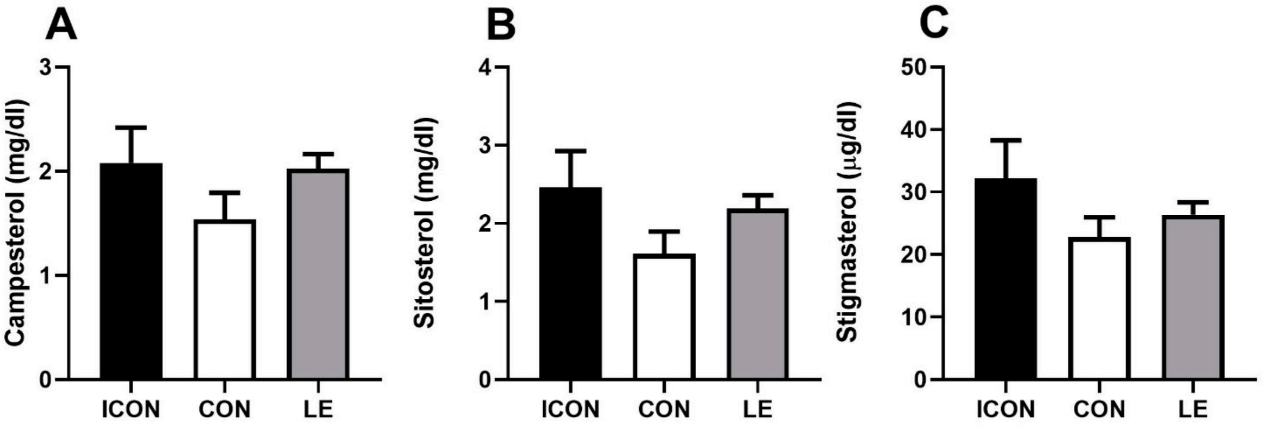

Figure S1. Effects of lemon flavonoid extract Eriomin (LE) on the concentration of plant sterols campesterol (A), sitosterol (B), and stigmasterol (C) in serum in old-aged male rats. The groups are abbreviated as follows: intact control (ICON), sunflower oil-treated control (CON), and lemon extract-treated group (LE). Data are expressed as mean ± SEM (n=7/group); comparisons between the CON and the study groups were not statistically significant (one-way ANOVA followed by Dunnet's post hoc).

Table S3. Listing results of in silico analysis using ADMETlab 3.0 for eriocitrin

Additionally, the corresponding relationships of the three labels are as follows: ● excellent; ● medium; ● poor;

Metabolism ERIOCITRIN

Property	Value	Decision	Comment
CYP1A2 inhibitor	0.0	●	■ Category 1: Inhibitor; Category 0: Non-inhibitor; ■ The output value is the probability of being inhibitor.
CYP1A2 substrate	0.0	●	■ Category 1: Substrate; Category 0: Non-substrate; ■ The output value is the probability of being substrate.
CYP2C19 inhibitor	0.0	●	■ Category 1: Inhibitor; Category 0: Non-inhibitor; ■ The output value is the probability of being inhibitor.
CYP2C19 substrate	0.0	●	■ Category 1: Substrate; Category 0: Non-substrate; ■ The output value is the probability of being substrate.
CYP2C9 inhibitor	0.0	●	■ Category 1: Inhibitor; Category 0: Non-inhibitor; ■ The output value is the probability of being inhibitor.
CYP2C9 substrate	0.001	●	■ Category 1: Substrate; Category 0: Non-substrate; ■ The output value is the probability of being substrate.
CYP2D6 inhibitor	0.0	●	■ Category 1: Inhibitor; Category 0: Non-inhibitor; ■ The output value is the probability of being inhibitor.
CYP2D6 substrate	0.0	●	■ Category 1: Substrate; Category 0: Non-substrate; ■ The output value is the probability of being substrate.
CYP3A4 inhibitor	0.001	●	■ Category 1: Inhibitor; Category 0: Non-inhibitor; ■ The output value is the probability of being inhibitor.
CYP3A4 substrate	0.0	●	■ Category 1: Substrate; Category 0: Non-substrate; ■ The output value is the probability of being substrate.
CYP2B6 inhibitor	0.0	●	■ Category 1: Inhibitor; Category 0: Non-inhibitor; ■ The output value is the probability of being inhibitor.
CYP2B6 substrate	0.0	●	■ Category 1: Substrate; Category 0: Non-substrate; ■ The output value is the probability of being substrate.
CYP2C8 inhibitor	0.081	●	■ Category 1: Inhibitor; Category 0: Non-inhibitor; ■ The output value is the probability of being inhibitor.
HLM Stability	0.005	●	■ human liver microsomal (HLM) stability ■ Category 0: stable+ (HLM > 30 min); Category 1: unstable- (HLM ≤ 30 min). The output value is the probability of human liver microsomal instability, where a value closer to 1 indicates a higher likelihood of instability. The range is between 0 and 1.

Toxicity

ERIOCITRIN

Property	Value	Decision	Comment
hERG Blockers	0.062	●	■ Molecules with IC50 ≤10μM or ≥50% inhibition at 10 μM were classified as hERG+ (Category 1), ■ while molecules with IC50 >10μM or < 50% inhibition at 10μM were classified as hERG - (Category 0). ■ The output value is the probability of being hERG+, within the range of 0 to 1.
hERG Blockers (10um)	0.677	●	■ Molecules with IC50 ≤10 μM are classified as hERG+ (Category 1), ■ and molecules with IC50 > 10μM are classified as hERG- (Category 0). ■ The output value is the probability of being hERG+, within the range of 0 to 1.
DILI	0.864	●	■ Drug Induced Liver Injury. ■ Category 1: drugs with a high risk of DILI; ■ Category 0: drugs with no risk of DILI. ■ The output value is the probability of being toxic.
AMES Muta genicity	0.872	●	■ AMES Toxicity ■ Category 1: Ames positive(+); ■ Category 0: Ames negative(-); ■ The output value is the probability of being toxic.
Rat Oral Acute Toxicity	0.518	●	■ Rat Oral Acute Toxicity. ■ Category 0: low-toxicity, > 500 mg/kg; ■ Category 1: high-toxicity; < 500 mg/kg. ■ The output value is the probability of being toxic, within the range of 0 to 1.
FDAMDD	0.402	●	■ FDA Maximum (Recommended) Daily Dose. ■ Category 1: FDAMDD (+); ■ Category 0: FDAMDD (-); The output value is the probability of being positive.
Skin Sensiti zation	0.386	●	■ Category 1: Sensitizer; ■ Category 0: Non-sensitizer. ■ The output value is the probability of being toxic, within the range of 0 to 1.
Carcinogeni city	0.111	●	■ Category 1: carcinogens; ■ Category 0: non-carcinogens; ■ The output value is the probability of being toxic.

Eye Corrosion	0.0	●	■ Eye Corrosion ■ Category 1: corrosives; Category 0: noncorrosives; ■ The output value is the probability of being corrosives.
Eye Irritation	0.024	●	■ Eye Irritation ■ Category 1: irritants; Category 0: nonirritants; ■ The output value is the probability of being irritants.
Respiratory	0.024	●	■ Category 1: respiratory toxicants; ■ Category 0: non-respiratory toxicants. ■ The output value is the probability of being toxic, within the range of 0 to 1.
Human Hep atotoxicity	0.475	●	■ Human Hepatotoxicity ■ Category 1: H-HT positive(+); ■ Category 0: H-HT negative(-); ■ The output value is the probability of being toxic.
Drug-induce d Nephrotox icity	0.08	●	■ Category 0: non-nephrotoxic (-); ■ Category 1: nephrotoxic (+). ■ The output value is the probability of being nephrotoxic (+), within the range of 0 to 1.
Ototoxicity	0.994	●	■ Category 0: non-ototoxicity (-); ■ Category 1: ototoxicity (+). ■ The output value is the probability of being ototoxicity (+), within the range of 0 to 1.
Hematotoxic ity	0.024	●	■ Category 0: non-hematotoxicity (-); ■ Category 1: hematotoxicity (+). ■ The output value is the probability of being hematotoxicity (+), within the range of 0 to 1.
Genotoxicity	0.823	●	■ Category 0: non-Genotoxicity (-); ■ Category 1: Genotoxicity (+). ■ The output value is the probability of being ototoxicity (+), within the range of 0 to 1.
RPMI-8226 Immunitoxici ty	0.062	●	■ Category 0: non-cytotoxicity (-); ■ Category 1: cytotoxicity (+). ■ The output value is the probability of being ototoxicity (+), within the range of 0 to 1.
A549 Cytotoxicity	0.665	●	■ Category 0: non-cytotoxicity (-); ■ Category 1: cytotoxicity (+). ■ The output value is the probability of being ototoxicity (+), within the range of 0 to 1.
Hek293 Cytotoxicity	0.848	●	■ Category 0: non-cytotoxicity (-); ■ Category 1: cytotoxicity (+). ■ The output value is the probability of being ototoxicity (+), within the range of 0 to 1.
Drug-induce d Neurotoxi city	0.084	●	■ Category 0: non-neurotoxic (-); ■ Category 1: neurotoxic (+). ■ The output value is the probability of being neurotoxic (+), within the range of 0 to 1.

ADMETlab 3.0

Table S4. Listing results of in silico analysis using ADMETlab 3.0 for naringin

Metabolism NARINGIN

Property	Value	Decision	Comment
CYP1A2 inhibitor	0.0	●	■ Category 1: Inhibitor; Category 0: Non-inhibitor; ■ The output value is the probability of being inhibitor.
CYP1A2 substrate	0.0	●	■ Category 1: Substrate; Category 0: Non-substrate; ■ The output value is the probability of being substrate.
CYP2C19 inhibitor	0.0	●	■ Category 1: Inhibitor; Category 0: Non-inhibitor; ■ The output value is the probability of being inhibitor.
CYP2C19 substrate	0.0	●	■ Category 1: Substrate; Category 0: Non-substrate; ■ The output value is the probability of being substrate.
CYP2C9 inhibitor	0.0	●	■ Category 1: Inhibitor; Category 0: Non-inhibitor; ■ The output value is the probability of being inhibitor.
CYP2C9 substrate	0.105	●	■ Category 1: Substrate; Category 0: Non-substrate; ■ The output value is the probability of being substrate.
CYP2D6 inhibitor	0.0	●	■ Category 1: Inhibitor; Category 0: Non-inhibitor; ■ The output value is the probability of being inhibitor.
CYP2D6 substrate	0.0	●	■ Category 1: Substrate; Category 0: Non-substrate; ■ The output value is the probability of being substrate.
CYP3A4 inhibitor	0.001	●	■ Category 1: Inhibitor; Category 0: Non-inhibitor; ■ The output value is the probability of being inhibitor.
CYP3A4 substrate	0.0	●	■ Category 1: Substrate; Category 0: Non-substrate; ■ The output value is the probability of being substrate.
CYP2B6 inhibitor	0.0	●	■ Category 1: Inhibitor; Category 0: Non-inhibitor; ■ The output value is the probability of being inhibitor.
CYP2B6 substrate	0.0	●	■ Category 1: Substrate; Category 0: Non-substrate; ■ The output value is the probability of being substrate.
CYP2C8 inhibitor	0.037	●	■ Category 1: Inhibitor; Category 0: Non-inhibitor; ■ The output value is the probability of being inhibitor.
HLM Stability	0.0	●	■ human liver microsomal (HLM) stability ■ Category 0: stable+ (HLM > 30 min); Category 1: unstable- (HLM ≤ 30 min). The output value is the probability of human liver microsomal instability, where a value closer to 1 indicates a higher likelihood of instability. The range is between 0 and 1.

Toxicity NARINGIN

Property	Value	Decision	Comment
hERG Blockers	0.067	●	■ Molecules with IC50 ≤10μM or ≥50% inhibition at 10 μM were classified as hERG+ (Category 1), ■ while molecules with IC50 >10μM or < 50% inhibition at 10μM were classified as hERG - (Category 0). ■ The output value is the probability of being hERG+, within the range of 0 to 1.
hERG Blockers (10um)	0.446	●	■ Molecules with IC50 ≤10 μM are classified as hERG+ (Category 1), ■ and molecules with IC50 > 10μM are classified as hERG- (Category 0). ■ The output value is the probability of being hERG+, within the range of 0 to 1.
DILI	0.736	●	■ Drug Induced Liver Injury. ■ Category 1: drugs with a high risk of DILI; ■ Category 0: drugs with no risk of DILI. ■ The output value is the probability of being toxic.
AMES Muta genicity	0.774	●	■ AMES Toxicity ■ Category 1: Ames positive(+); ■ Category 0: Ames negative(-); ■ The output value is the probability of being toxic.
Rat Oral Acute Toxicity	0.426	●	■ Rat Oral Acute Toxicity. ■ Category 0: low-toxicity, > 500 mg/kg; ■ Category 1: high-toxicity; < 500 mg/kg. ■ The output value is the probability of being toxic, within the range of 0 to 1.
FDAMDD	0.32	●	■ FDA Maximum (Recommended) Daily Dose. ■ Category 1: FDAMDD (+); ■ Category 0: FDAMDD (-); The output value is the probability of being positive.
Skin Sensiti zation	0.189	●	■ Category 1: Sensitizer; ■ Category 0: Non-sensitizer. ■ The output value is the probability of being toxic, within the range of 0 to 1.
Carcinogeni city	0.187	●	■ Category 1: carcinogens; ■ Category 0: non-carcinogens; ■ The output value is the probability of being toxic.

Eye Corrosion	0.0	●	■ Eye Corrosion ■ Category 1: corrosives; Category 0: noncorrosives; ■ The output value is the probability of being corrosives.
Eye Irritation	0.059	●	■ Eye Irritation ■ Category 1: irritants; Category 0: nonirritants; ■ The output value is the probability of being irritants.
Respiratory	0.038	●	■ Category 1: respiratory toxicants; ■ Category 0: non-respiratory toxicants. ■ The output value is the probability of being toxic, within the range of 0 to 1.
Human Hep atotoxicity	0.689	●	■ Human Hepatotoxicity ■ Category 1: H-HT positive(+); ■ Category 0: H-HT negative(-); ■ The output value is the probability of being toxic.
Drug-induce d Nephrotox icity	0.287	●	■ Category 0: non-nephrotoxic (-); ■ Category 1: nephrotoxic (+). ■ The output value is the probability of being nephrotoxic (+), within the range of 0 to 1.
Ototoxicity	0.987	●	■ Category 0: non-ototoxicity (-); ■ Category 1: ototoxicity (+). ■ The output value is the probability of being ototoxicity (+), within the range of 0 to 1.
Hematotoxic ity	0.061	●	■ Category 0: non-hematotoxicity (-); ■ Category 1: hematotoxicity (+). ■ The output value is the probability of being hematotoxicity (+), within the range of 0 to 1.
Genotoxicity	0.561	●	■ Category 0: non-Genotoxicity (-); ■ Category 1: Genotoxicity (+). ■ The output value is the probability of being ototoxicity (+), within the range of 0 to 1.
RPMI-8226 Immunitoxici ty	0.123	●	■ Category 0: non-cytotoxicity (-); ■ Category 1: cytotoxicity (+). ■ The output value is the probability of being ototoxicity (+), within the range of 0 to 1.
A549 Cytotoxicity	0.223	●	■ Category 0: non-cytotoxicity (-); ■ Category 1: cytotoxicity (+). ■ The output value is the probability of being ototoxicity (+), within the range of 0 to 1.
Hek293 Cytotoxicity	0.883	●	■ Category 0: non-cytotoxicity (-); ■ Category 1: cytotoxicity (+). ■ The output value is the probability of being ototoxicity (+), within the range of 0 to 1.
Drug-induce d Neurotoxi city	0.321	●	■ Category 0: non-neurotoxic (-); ■ Category 1: neurotoxic (+). ■ The output value is the probability of being neurotoxic (+), within the range of 0 to 1.

Table S5. Listing results of in silico analysis using ADMETlab 3.0 for hesperidin

Metabolism HESPERIDIN

Property	Value	Decision	Comment
CYP1A2 inhibitor	0.0	●	■ Category 1: Inhibitor; Category 0: Non-inhibitor; ■ The output value is the probability of being inhibitor.
CYP1A2 substrate	0.0	●	■ Category 1: Substrate; Category 0: Non-substrate; ■ The output value is the probability of being substrate.
CYP2C19 inhibitor	0.0	●	■ Category 1: Inhibitor; Category 0: Non-inhibitor; ■ The output value is the probability of being inhibitor.
CYP2C19 substrate	0.0	●	■ Category 1: Substrate; Category 0: Non-substrate; ■ The output value is the probability of being substrate.
CYP2C9 inhibitor	0.0	●	■ Category 1: Inhibitor; Category 0: Non-inhibitor; ■ The output value is the probability of being inhibitor.
CYP2C9 substrate	0.161	●	■ Category 1: Substrate; Category 0: Non-substrate; ■ The output value is the probability of being substrate.
CYP2D6 inhibitor	0.0	●	■ Category 1: Inhibitor; Category 0: Non-inhibitor; ■ The output value is the probability of being inhibitor.
CYP2D6 substrate	0.0	●	■ Category 1: Substrate; Category 0: Non-substrate; ■ The output value is the probability of being substrate.
CYP3A4 inhibitor	0.0	●	■ Category 1: Inhibitor; Category 0: Non-inhibitor; ■ The output value is the probability of being inhibitor.
CYP3A4 substrate	0.049	●	■ Category 1: Substrate; Category 0: Non-substrate; ■ The output value is the probability of being substrate.
CYP2B6 inhibitor	0.0	●	■ Category 1: Inhibitor; Category 0: Non-inhibitor; ■ The output value is the probability of being inhibitor.
CYP2B6 substrate	0.0	●	■ Category 1: Substrate; Category 0: Non-substrate; ■ The output value is the probability of being substrate.
CYP2C8 inhibitor	0.069	●	■ Category 1: Inhibitor; Category 0: Non-inhibitor; ■ The output value is the probability of being inhibitor.
HLM Stability	0.003	●	■ human liver microsomal (HLM) stability ■ Category 0: stable+ (HLM > 30 min); Category 1: unstable- (HLM ≤ 30 min). The output value is the probability of human liver microsomal instability, where a value closer to 1 indicates a higher likelihood of instability. The range is between 0 and 1.

Toxicity

HESPERIDIN

Property	Value	Decision	Comment
hERG Blockers	0.066	🟢	■ Molecules with IC50 ≤10μM or ≥50% inhibition at 10 μM were classified as hERG+ (Category 1), ■ while molecules with IC50 >10μM or < 50% inhibition at 10μM were classified as hERG - (Category 0). ■ The output value is the probability of being hERG+, within the range of 0 to 1.
hERG Blockers (10um)	0.565	🟠	■ Molecules with IC50 ≤10 μM are classified as hERG+ (Category 1), ■ and molecules with IC50 > 10μM are classified as hERG- (Category 0). ■ The output value is the probability of being hERG+, within the range of 0 to 1.
DILI	0.907	🔴	■ Drug Induced Liver Injury. ■ Category 1: drugs with a high risk of DILI; ■ Category 0: drugs with no risk of DILI. ■ The output value is the probability of being toxic.
AMES Muta genicity	0.86	🔴	■ AMES Toxicity ■ Category 1: Ames positive(+); ■ Category 0: Ames negative(-); ■ The output value is the probability of being toxic.
Rat Oral Acute Toxicity	0.501	🟠	■ Rat Oral Acute Toxicity. ■ Category 0: low-toxicity, > 500 mg/kg; ■ Category 1: high-toxicity; < 500 mg/kg. ■ The output value is the probability of being toxic, within the range of 0 to 1.
FDAMDD	0.365	🟠	■ FDA Maximum (Recommended) Daily Dose. ■ Category 1: FDAMDD (+); ■ Category 0: FDAMDD (-); The output value is the probability of being positive.
Skin Sensiti zation	0.055	🟢	■ Category 1: Sensitizer; ■ Category 0: Non-sensitizer. ■ The output value is the probability of being toxic, within the range of 0 to 1.
Carcinogeni city	0.186	🟢	■ Category 1: carcinogens; ■ Category 0: non-carcinogens; ■ The output value is the probability of being toxic.
Eye Corrosion	0.0	🟢	■ Eye Corrosion ■ Category 1: corrosives; Category 0: noncorrosives; ■ The output value is the probability of being corrosives.
Eye Irritation	0.012	🟢	■ Eye Irritation ■ Category 1: irritants; Category 0: nonirritants; ■ The output value is the probability of being irritants.
Respiratory	0.062	🟢	■ Category 1: respiratory toxicants; ■ Category 0: non-respiratory toxicants. ■ The output value is the probability of being toxic, within the range of 0 to 1.
Human Hep atotoxicity	0.49	🟠	■ Human Hepatotoxicity ■ Category 1: H-HT positive(+); ■ Category 0: H-HT negative(-); ■ The output value is the probability of being toxic.
Drug-induce d Nephrotox icity	0.206	🟢	■ Category 0: non-nephrotoxic (-); ■ Category 1: nephrotoxic (+). ■ The output value is the probability of being nephrotoxic (+), within the range of 0 to 1.
Ototoxicity	0.983	🔴	■ Category 0: non-ototoxicity (-); ■ Category 1: ototoxicity (+). ■ The output value is the probability of being ototoxicity (+), within the range of 0 to 1.
Hematotoxic ity	0.05	🟢	■ Category 0: non-hematotoxicity (-); ■ Category 1: hematotoxicity (+). ■ The output value is the probability of being hematotoxicity (+), within the range of 0 to 1.
Genotoxicity	0.593	🟠	■ Category 0: non-Genotoxicity (-); ■ Category 1: Genotoxicity (+). ■ The output value is the probability of being ototoxicity (+), within the range of 0 to 1.
RPMI-8226 Immunitoxici ty	0.211	🟢	■ Category 0: non-cytotoxicity (-); ■ Category 1: cytotoxicity (+). ■ The output value is the probability of being ototoxicity (+), within the range of 0 to 1.
A549 Cytotoxicity	0.296	🟢	■ Category 0: non-cytotoxicity (-); ■ Category 1: cytotoxicity (+). ■ The output value is the probability of being ototoxicity (+), within the range of 0 to 1.
Hek293 Cytotoxicity	0.891	🔴	■ Category 0: non-cytotoxicity (-); ■ Category 1: cytotoxicity (+). ■ The output value is the probability of being ototoxicity (+), within the range of 0 to 1.
Drug-induce d Neurotoxi city	0.318	🟠	■ Category 0: non-neurotoxic (-); ■ Category 1: neurotoxic (+). ■ The output value is the probability of being neurotoxic (+), within the range of 0 to 1.

Table S6. Listing results of in silico analysis using ADMETlab 3.0 for didymin

Metabolism DIDYMIN

Property	Value	Decision	Comment
CYP1A2 inhibitor	0.0	●	■ Category 1: Inhibitor; Category 0: Non-inhibitor; ■ The output value is the probability of being inhibitor.
CYP1A2 substrate	0.0	●	■ Category 1: Substrate; Category 0: Non-substrate; ■ The output value is the probability of being substrate.
CYP2C19 inhibitor	0.0	●	■ Category 1: Inhibitor; Category 0: Non-inhibitor; ■ The output value is the probability of being inhibitor.
CYP2C19 substrate	0.001	●	■ Category 1: Substrate; Category 0: Non-substrate; ■ The output value is the probability of being substrate.
CYP2C9 inhibitor	0.0	●	■ Category 1: Inhibitor; Category 0: Non-inhibitor; ■ The output value is the probability of being inhibitor.
CYP2C9 substrate	0.056	●	■ Category 1: Substrate; Category 0: Non-substrate; ■ The output value is the probability of being substrate.
CYP2D6 inhibitor	0.0	●	■ Category 1: Inhibitor; Category 0: Non-inhibitor; ■ The output value is the probability of being inhibitor.
CYP2D6 substrate	0.0	●	■ Category 1: Substrate; Category 0: Non-substrate; ■ The output value is the probability of being substrate.
CYP3A4 inhibitor	0.0	●	■ Category 1: Inhibitor; Category 0: Non-inhibitor; ■ The output value is the probability of being inhibitor.
CYP3A4 substrate	0.001	●	■ Category 1: Substrate; Category 0: Non-substrate; ■ The output value is the probability of being substrate.
CYP2B6 inhibitor	0.0	●	■ Category 1: Inhibitor; Category 0: Non-inhibitor; ■ The output value is the probability of being inhibitor.
CYP2B6 substrate	0.0	●	■ Category 1: Substrate; Category 0: Non-substrate; ■ The output value is the probability of being substrate.
CYP2C8 inhibitor	0.039	●	■ Category 1: Inhibitor; Category 0: Non-inhibitor; ■ The output value is the probability of being inhibitor.
HLM Stability	0.002	●	■ human liver microsomal (HLM) stability ■ Category 0: stable+ (HLM > 30 min); Category 1: unstable- (HLM ≤ 30 min). The output value is the probability of human liver microsomal instability, where a value closer to 1 indicates a higher likelihood of instability. The range is between 0 and 1.

Toxicity

DIDYMIN

Property	Value	Decision	Comment
hERG Blockers	0.124	🟢	■ Molecules with IC50 ≤10μM or ≥50% inhibition at 10 μM were classified as hERG+ (Category 1),<br>■ while molecules with IC50 >10μM or < 50% inhibition at 10μM were classified as hERG - (Category 0).<br>■ The output value is the probability of being hERG+, within the range of 0 to 1.
hERG Blockers (10um)	0.578	🟠	■ Molecules with IC50 ≤10 μM are classified as hERG+ (Category 1),<br>■ and molecules with IC50 > 10μM are classified as hERG- (Category 0).<br>■ The output value is the probability of being hERG+, within the range of 0 to 1.
DILI	0.921	🔴	■ Drug Induced Liver Injury.<br>■ Category 1: drugs with a high risk of DILI;<br>■ Category 0: drugs with no risk of DILI.<br>■ The output value is the probability of being toxic.
AMES Muta genicity	0.889	🔴	■ AMES Toxicity<br>■ Category 1: Ames positive(+);<br>■ Category 0: Ames negative(-);<br>■ The output value is the probability of being toxic.
Rat Oral Acute Toxicity	0.479	🟠	■ Rat Oral Acute Toxicity.<br>■ Category 0: low-toxicity, > 500 mg/kg;<br>■ Category 1: high-toxicity; < 500 mg/kg.<br>■ The output value is the probability of being toxic, within the range of 0 to 1.
FDAMDD	0.324	🟠	■ FDA Maximum (Recommended) Daily Dose.<br>■ Category 1: FDAMDD (+);<br>■ Category 0: FDAMDD (-);<br>The output value is the probability of being positive.
Skin Sensiti zation	0.03	🟢	■ Category 1: Sensitizer;<br>■ Category 0: Non-sensitizer.<br>■ The output value is the probability of being toxic, within the range of 0 to 1.
Carcinogeni city	0.194	🟢	■ Category 1: carcinogens;<br>■ Category 0: non-carcinogens;<br>■ The output value is the probability of being toxic.
Eye Corrosion	0.0	🟢	■ Eye Corrosion<br>■ Category 1: corrosives;<br>Category 0: noncorrosives;<br>■ The output value is the probability of being corrosives.
Eye Irritation	0.009	🟢	■ Eye Irritation<br>■ Category 1: irritants;<br>Category 0: nonirritants;<br>■ The output value is the probability of being irritants.
Respiratory	0.049	🟢	■ Category 1: respiratory toxicants;<br>■ Category 0: non-respiratory toxicants.<br>■ The output value is the probability of being toxic, within the range of 0 to 1.
Human Hep atotoxicity	0.544	🟠	■ Human Hepatotoxicity<br>■ Category 1: H-HT positive(+);<br>■ Category 0: H-HT negative(-);<br>■ The output value is the probability of being toxic.
Drug-induce d Nephrotox icity	0.403	🟠	■ Category 0: non-nephrotoxic (-);<br>■ Category 1: nephrotoxic (+).<br>■ The output value is the probability of being nephrotoxic (+), within the range of 0 to 1.
Ototoxicity	0.986	🔴	■ Category 0: non-ototoxicity (-);<br>■ Category 1: ototoxicity (+).<br>■ The output value is the probability of being ototoxicity (+), within the range of 0 to 1.
Hematotoxic ity	0.086	🟢	■ Category 0: non-hematotoxicity (-);<br>■ Category 1: hematotoxicity (+).<br>■ The output value is the probability of being hematotoxicity (+), within the range of 0 to 1.
Genotoxicity	0.413	🟠	■ Category 0: non-Genotoxicity (-);<br>■ Category 1: Genotoxicity (+).<br>■ The output value is the probability of being ototoxicity (+), within the range of 0 to 1.
RPMI-8226 Immunitoxici ty	0.185	🟢	■ Category 0: non-cytotoxicity (-);<br>■ Category 1: cytotoxicity (+).<br>■ The output value is the probability of being ototoxicity (+), within the range of 0 to 1.
A549 Cytotoxicity	0.225	🟢	■ Category 0: non-cytotoxicity (-);<br>■ Category 1: cytotoxicity (+).<br>■ The output value is the probability of being ototoxicity (+), within the range of 0 to 1.
Hek293 Cytotoxicity	0.876	🔴	■ Category 0: non-cytotoxicity (-);<br>■ Category 1: cytotoxicity (+).<br>■ The output value is the probability of being ototoxicity (+), within the range of 0 to 1.
Drug-induce d Neurotoxi city	0.472	🟠	■ Category 0: non-neurotoxic (-);<br>■ Category 1: neurotoxic (+).<br>■ The output value is the probability of being neurotoxic (+), within the range of 0 to 1.

ADMETlab 3.0
